# The *Drosophila* Citrate Lyase Is Required for Cell Division during Spermatogenesis

**DOI:** 10.3390/cells9010206

**Published:** 2020-01-14

**Authors:** Maria Laura Di Giorgio, Patrizia Morciano, Elisabetta Bucciarelli, Antonella Porrazzo, Francesca Cipressa, Sara Saraniero, Diana Manzi, Yikang S. Rong, Giovanni Cenci

**Affiliations:** 1 Dipartimento di Biologia e Biotecnologie “C. Darwin”, SAPIENZA Università di Roma, P.le A. Moro 5, 00185 Rome, Italy; 2 INFN-Laboratori Nazionali del Gran Sasso, I-67100 Assergi (L’Aquila), Italy; 3 Istituto di Biologia e Patologia Molecolari (IBPM) del CNR, 00185 Rome, Italy; 4 Istituto Pasteur, Fondazione Cenci Bolognetti, 00185 Rome, Italy; 5 State Key Laboratory of Bio-Control, School of Life Sciences, Sun Yat-sen University, Guangzhou 510275, China

**Keywords:** ACL, *Drosophila*, male meiosis, spermatogenesis

## Abstract

The *Drosophila melanogaster DmATPCL* gene encodes for the human ATP Citrate Lyase (ACL) ortholog, a metabolic enzyme that from citrate generates glucose-derived Acetyl-CoA, which fuels central biochemical reactions such as the synthesis of fatty acids, cholesterol and acetylcholine, and the acetylation of proteins and histones. We had previously reported that, although loss of *Drosophila* ATPCL reduced levels of Acetyl-CoA, unlike its human counterpart, it does not affect global histone acetylation and gene expression, suggesting that its role in histone acetylation is either partially redundant in *Drosophila* or compensated by alternative pathways. Here, we describe that depletion of DmATPCL affects spindle organization, cytokinesis, and fusome assembly during male meiosis, revealing an unanticipated role for DmATPCL during spermatogenesis. We also show that *DmATPCL* mutant meiotic phenotype is in part caused by a reduction of fatty acids, but not of triglycerides or cholesterol, indicating that DmATPCL-derived Acetyl-CoA is predominantly devoted to the biosynthesis of fatty acids during spermatogenesis. Collectively, our results unveil for the first time an involvement for DmATPCL in the regulation of meiotic cell division, which is likely conserved in human cells.

## 1. Introduction

The highly conserved ATP citrate lyase (ACL) catalyzes the ATP-dependent and coenzyme A (CoA)-dependent conversion of mitochondria-exported citrate to oxaloacetate and the Acetyl-CoA [1]. The latter high-energy biosynthetic precursor fuels central biochemical reactions such as the synthesis of fatty acids, cholesterol and acetylcholine, and the acetylation of proteins and histones. The abundance and distribution of Acetyl-CoA in different cellular compartments may vary considerably depending on several physiological and pathological conditions. In the mitochondria, as a product of pyruvate dehydrogenase, Acetyl-CoA is the initiating metabolite of the tricarboxylic acid (TCA) cycle and used for the generation of ATP and the synthesis of amino acid carbon skeletons. It is also the final product of the mitochondrial fatty acid β-oxidation and the catabolism of branched amino acids.

ACL resides mainly in the endoplasmic reticulum in mammalian cells [1], but it has been found also in the nucleus, where it is required for histone acetylation, gene expression regulation, and more recently in DNA repair [2,3]. However, given its fundamental role in the production of cytosolic Acetyl-CoA, ACL could be involved in several, as yet unidentified, physiological processes. Recently, high-resolution crystal structures of bacterial, archaeal, and human ACL have been released and unmask a fundamental evolutionary relationship that links today enzyme to an ancestral lyase module, marking a key step in the evolution of metabolism on Earth [4].

ACL is reported to play a role in different human pathologies. Recent works demonstrated that ACL acts as an epigenetic regulator to promote obesity-related kidney injury [5]. In addition, bioinformatics studies identified ACL as hub gene in the molecular process of type 2 diabetes, and therefore a potential target in the molecular mechanism of the disease [6]. Moreover, a large number of evidence indicates that ACL is up-regulated or activated in lung, prostate, bladder, breast, liver, stomach, and colon cancers [7,8], and its inhibition by chemical inhibitors or RNAi dramatically suppresses proliferation of certain types of tumor cells in vitro and in vivo, making ACL a promising therapeutic target to counteract cancer growth and progression [9,10,11].

*Drosophila* genome encodes one ACL ortholog, ATPCL, which shares 70% of identity with its human counterpart [12,13]. We previously showed that although depletion of DmATPCL reduced levels of Acetyl CoA in larvae and adult flies, unlike its human counterpart, it does not affect global histone acetylation and gene expression. Nevertheless, DmATPCL depletion led to a moderate chromosome breakage frequency that increased in the presence of mutations in the mitochondrial citrate carrier SLC25A. This suggests that in mitotic cells, while DmATPCL has a dispensable role in histone acetylation, it prevents massive chromosome fragmentation when citrate efflux is altered [12]. Here, we show that *DmATPCL* mutant testes display irregular spindle organization, frequent multinucleated spermatids, and abnormal fusome in primary spermatocyte cysts, indicating that an impairment of DmATPCL function affects male spermatogenesis at different levels. Interestingly, *DmATPCL* mutant meiotic phenotype is caused by a reduction of fatty acid, but not a decrease in protein acetylation, suggesting that DmATPCL-derived Acetyl-CoA is predominantly devoted to the biosynthesis of fatty acids during spermatogenesis. Collectively, our results, obtained in a well-established model organism for human biology, unveil an unanticipated involvement for DmATPCL in the regulation of meiotic cell division and male fertility, which is likely conserved in human cells.

## 2. Materials and Methods

### 2.1. Drosophila Strains and Crosses

The insertion lines *DmATPCL^01466^* and *DmATPCL^DG23402^*, as well as the *Df(2R)Exel7138* that uncovers *DmATPCL*, were obtained from the Bloomington Stock Center and were balanced over *CyTb*, as previously described [12]. Flies were raised on standard corn-meal food. For fatty acids feeding, both wild-type and mutant flies were raised on normal corn-meal food supplemented with 0.5% oleic and arachidonic acids. F1 larvae and adults were then analyzed. 

### 2.2. Chromosome Cytology, Immunostaining, and Microscopy

Fixation and F-actin staining of *Drosophila* testes were performed as described in [14]. Fixation for the other immunostainings was performed as previously described [15,16]. The primary antibodies and the dilutions (in PBS) used were as follows: Anti-tub (1:1000) (Sigma-Aldrich, St. Louis, MO, USA), anti-Pav (1:100) [17,18], anti-Spd2 (1:5000) [19], anti-Feo (1:50) [20], anti-HTS (IBI) (1:5) (Hybridoma Bank, The University of Iowa, IA, USA) [21], anti-anillin (1:1000) [22]. The secondary antibody incubation was performed using both the FITC-conjugated anti-mouse IgG+IgM (1:20 in PBS; Jackson ImmunoResearch Laboratories, Cambridge, UK) and Alexa 555-conjugated anti-rabbit IgG (1:300 in PBS; Molecular Probes, Eugene, OR, USA) for 2 h at room temperature. Slides were then mounted in Vectashield medium H-1200 with DAPI (Vector Laboratories, Burlingame, CA, USA) to stain DNA and reduce fluorescence fading.

Slides with mitotic chromosome preparations and fixed testes were analyzed using a Zeiss Axioplan epifluorescence microscope (Carl Zeiss, Obezkochen, Germany), equipped with a cooled CCD camera (Photometrics, Woburn, MA, USA). Gray-scale digital images were collected separately, converted to Photoshop format, pseudocolored, and merged.

### 2.3. RNA Extraction, cDNA Amplification, and qPCR

Total RNA was isolated from larval testes (50 testes/sample) using TRIzol (TRI Reagent^®^ SIGMA Life Science). RNA concentration and purity were measured at the NanoDrop 1000 Spectrophotometer (ThermoScientific, Whaltman, MA, USA) with the NanoDrop 1000 3.7.1 software. Genomic DNA was eliminated with Invitrogen™ DNase I, Amplification Grade (Carlsbad, CA, USA). The analysis of the expression levels of *DmATPCL* transcripts was carried out as previously described [12].

### 2.4. Western Blotting

To obtain testes extracts for the Western Blot analysis, larval testes were lysed in an ice-cold buffer containing 20 mM Hepes KOH pH 7.9, 1.5 mM MgCl_2_, 10 mM KCl, 420 mM NaCl, 30 mM NaF, 0.2 mM Na_3_Vo_4_, 25 mM β-glycerophosphate, 0.5 M PMSF, 0.1% NP40, 1× protease inhibitor cocktail (Roche, Basel, Switzerland). For immunoblotting, protein samples were resuspended in 1× Laemmli Buffer, run into SDS polyacrilammide gels, and electroblotted on a nitrocellulose membrane (Bio-Rad, Hercules, CA, USA) in a phosphate buffer containing 390 mM NaH_2_PO_4_ and 610 mM Na_2_HPO_4_. After blocking with 5% low-fat dry milk, the membrane was probed with appropriate primary antibody. Anti-rabbit or anti-mouse HRP-conjugated secondary antibodies (1:5000; GE Healthcare, Chicago, IL, USA) were used as secondary antibodies. The blots were developed by the ECL or ECL Plus method (Amersham Biosciences, Little Chalfont, UK) and signals detected with the ChemiDoc scanning system (BioRad). The antibody dilutions were: Anti-DmATPCL (1:500), anti-acTub (Sigma; 1:10,000), anti Ac-Lys (1:1000; Merck-Millipore, Burlington, MA, USA). 

### 2.5. Acetyl-CoA, Cholesterol, Free Fatty Acids, and Triglycerides Quantifications

For Acetyl CoA analyses, 30 mg larval testes from Oregon R and *DmATPCL* mutants were homogenized and deproteinized using perchloric acid. Quantification of Acetyl-CoA was performed using the PicoProbe Acetyl-CoA Fluorometric Assay kit (Biovision, San Franscisco, CA, USA). Triglycerides and levels were measured from 30 mg third instar larvae using the IL ILAB 600 Analyzer (Diamond Diagnostic, Holliston, MA, USA) with the Infinity Triglycerides Liquid Stable Reagent (Thermo Scientific). Levels of Free Fatty Acids were measured using the Free Fatty Acid Quantification Kit (Biovision). Cholesterol concentration from 30 first instar larvae was estimated with the Amplex Red Cholesterol assay kit (Invitrogen) following the manufacturer’s protocol. Briefly, 30 first instar larvae were washed three times in 0.7% NaCl, homogenized with 150 μL of reaction buffer, and lysates were cleared by centrifugation 5 min 5000 rpm at 4 °C. Fifty μL of each supernatant was aliquoted in triplicate in a 96 wells microplate along with 50 μL of cholesterol solutions at known concentrations for standards curve, 10 μM hydrogen peroxide as a reaction positive control and no-cholesterol control. Fifty μL of reaction enzymes mix was added to each microplate well, and reactions were incubated for 30 min at 37 °C protected from light. Fluorescence was measured using excitation 560 nm and detection at 590 nm, and background fluorescence from no-cholesterol control reaction has been subtracted from each value. 

## 3. Results and Discussion

### 3.1. The Drosophila ATP Cytrate Lyase Is Required for a Proper Male Meiosis

We have recently characterized two semi-lethal *DmATPCL* insertion mutant alleles, *DmATPCL^01466^* and *DmATPCL^DG23402^*. The corresponding insertions, that are located within the 5′ end of the gene at position –340 and –1167 upstream of the translation initiation site, respectively, resulted in a strong reduction (up to 80% compared to wild-type) of both *DmATPCL* transcripts and proteins [12]. Surprisingly, *DmATPCL^01466^* homozygotes and *DmATPCL^01466^/Df(2R)Exel7138* hemizygotes (*Df(2R)Exel7138* is a deficiency in the 52D1-52D12 polytene region that removes *DmATPCL*) as well as *DmATPCL ^DG23402^* homozygotes and hemizygotes. Neither affected histone acetylation nor showed obvious mitotic phenotype, suggesting that *Dm*ATPCL does not play a crucial role in mitosis or its function is redundant [12]. Nevertheless, all homozygous and/or hemizygous adults were male sterile, indicating that DmATPCL is required for a proper male spermatogenesis. The expression of a wild type *UAS ATPCL* transgene under the control of a *TubGal4* driver in *DmATPCL^DG23402^* mutant background rescued the male sterility phenotype, confirming that it resulted indeed from lesions in the *DmATPCL* gene.

qPCR analysis on testis RNA revealed that, analogously to the expression profile previously described [12], *DmATPCL* encodes the *DmATPCL-RG*, *-RF*, and *-RD/RE* annotated transcripts that generated almost identical DmATPCL proteins. However, differently from mitosis, -RG and -RF male meiotic transcripts are ~5 times more expressed compared to the mitotic transcripts (Supplementary Figure S1a), indicating that *DmATPCL* is more expressed during male meiosis/spermatogenesis than in mitosis. Western blot analysis using our guinea pig anti-DmATPCL polyclonal antibody [12] recognizes a band of expected size (~130 KDa) in testis extracts that significantly decreased in both *DmATPCL^01466^/Df(2R)Exel7138* and *DmATPCL^DG23402^/Df(2R)Exel7138* mutant combinations (Supplementary Figure S1b). Consistently with our previous observation in mitosis, the *DmATPCL^01466^/Df(2R)Exel7138* hemizygotes yielded to the strongest reduction (~80%) of expression of the DmATPCL protein, indicating that this mutant allele combination represents the most severe mutant condition (see also below). Interestingly, a commercial anti-ACLY antibody, that recognizes the human citrate lyase, also cross-reacted with DmATPCL leading to the same WB expression pattern observed with our anti-DmATPCL antibody (data not shown), thus confirming the high level of conservation between the two proteins.

However, WB analysis on testis extracts with antibodies that recognize either acetylated lysines or acetylated tubulin revealed no difference among mutant and wild-type testes in the expression pattern of these different classes of acetylated proteins (Supplementary Figure S1b), suggesting that, in agreement with our previous observations in mitosis, the reduction of Acetyl-CoA synthesis does not impact global lysine acetylation pattern. Yet, it cannot be ruled out that only a subset of specific acetylated lysines are affected by this Acetyl-CoA reduction. Further studies are necessary to address this question.

### 3.2. Loss of DmATPCL Affects Centrosome Organization and Meiotic Spindle Formation

**Figure 1:**
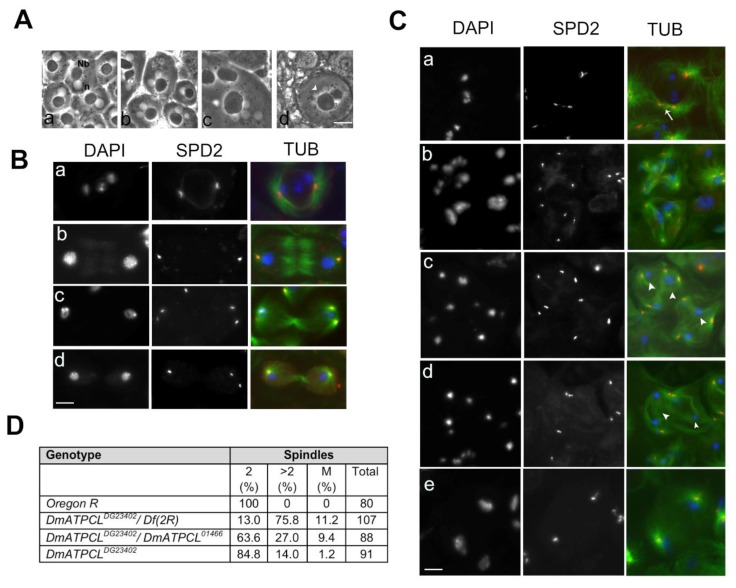
Loss of DmATPCL affects male meiosis. (**A**) Phase contrast images of onion stage spermatids from control (**Aa**) and *DmATPCL* mutant testes (**Ab**–**d**). Note that in *DmATPCL* mutant spermatids consists of a large nebenkern (Nb) associated to either two or four nuclei (n) in *DmATPCL ^DG23402^*/*DmATPCL ^DG23402^* combination (**Ab**,**c**) as well as to aneuploidy nuclei *DmATPCL ^DG23402^/Df* genotype ((**Ad**), white arrowhead). (**B**,**C**) Immunostaining with anti-Spd2 (red) and anti-tubulin (green) in wild-type (**B**) and *DmATPCL* mutant (**C**) dividing meiotic cells. Panels in B show examples of control cells in prometaphase I (**Ba**), anaphase I (**Bb**), telophase I (**Bc**), and anatelophase II (**Bd**). DNA has been counterstained with DAPI. Note that depletion of DmATPCL induces premature centriole disengagement in primary spermatocytes (arrow in **Ca**) that leads to the formation of multipolar spindles (**Cb**–**d**) in which chromosomes do not segregate properly (arrowheads). (**Ce**) Example of a dividing cell with an apparent normal bipolar spindle that lacks a central spindle and/or fails to contract but with daughter nuclei decondensed in a telophase state, indicating that central spindle fails to constrict during telophase. Bar = 10 μm. (**D**). Frequency of cells displaying irregular meiotic spindles. 2 = Spindles with 2 asters (normal); >2 = Spindles with more than 2 asters (multipolar); M = monopolar spindle; *Df(2R)* = *Df(2R)Exel7138*.

In vivo characterization revealed that *DmATPCL* mutant testes displayed aberrant spermatids containing mitochondrial derivatives (nebenkern) of variable size associated with nuclei that were also different in size and number, indicating defects of meiotic chromosome segregation and cytokinesis (Figure 1A). To understand the causes of these aberrant phenotypes, we first characterized meiotic divisions in different *DmATPCL* mutant alleles using antibodies against tubulin and centrosomal marker Spd2 [23,24]. Male meiotic cells were staged according to Cenci et al. (1994) [25]. In wild type, dividing spermatocytes in meiosis I displayed two prominent centrosomes (each containing a pair of centrioles) that formed bipolar meiotic spindles (Figure 1B). In contrast, about 76%, 27%, or 14% of all dividing cells from *DmATPCL^DG23402^/Df(2R)Exel7138*, *DmATPCL^DG23402^/DmATPCL^01466^*, and *DmATPCL^DG23402^* mutant males, respectively, displayed a variable number of centrosomal foci and assembled monopolar or multipolar spindles, suggesting a defect in centrosome structure (Figure 1C,D). We were unable to characterize at a cytological level the strongest *DmATPCL^01466^/Df(2R)Exel7138* hemizygous mutants, as they exhibited few and highly aberrant meiotic figures.

**Figure 2:**
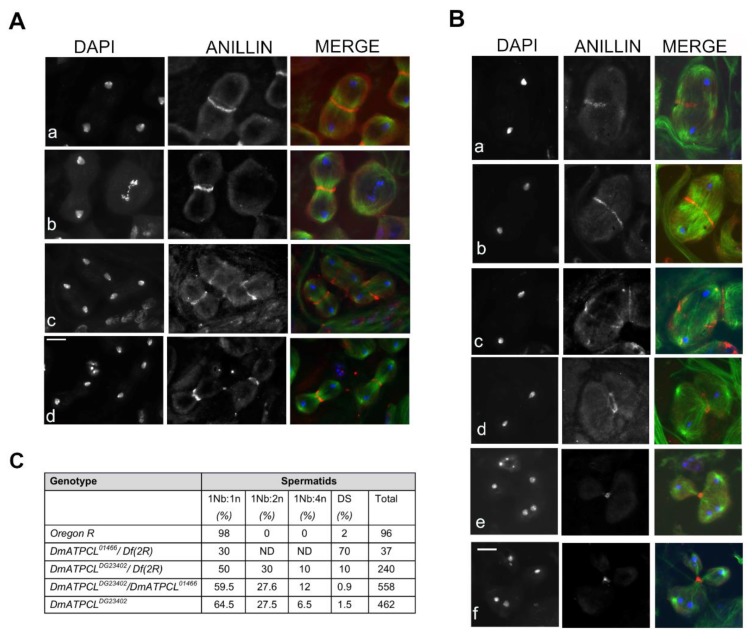
Depletion of DmATPCL impairs cytokinesis. Anti-anillin (red) and anti-tubulin (green) immunostaining in control (**A**) and *DmATPCL ^DG23402^* mutant allele (**B**). DNA is counterstained with DAPI. Note that in wild-type testes anillin starts to concentrate in a ring-shape configuration at the cell equator of dividing spermatocytes during anaphase and constricts during cytokinesis in telophase during both first (**Aa**,**b**) and second (**Ac**,**d**) meiotic divisions. In contrast, *DmATPCL ^DG23402^* mutant testes display cells with unconstrained central spindles during ana-telophase I (**Ba**–**c**) in which anillin localizes at the central spindle to eventually form a contractile ring that appeared fuzzy (**Ba**), remained unconstrained (**Bb**), and degenerates (**Bc**). In ana-telophase I cells with multipolar spindles (**Bd**–**f**) anillin still localizes at the equator of cells and participates to the formation of one single contractile ring that eventually completed cytokinesis, ultimately giving rise to irregular spermatids. Bar = 10 μm. (**C**) Frequency of irregular spermatids from different *DmATPCL* mutant combinations. 1Nb:1n = normal “onion stage” spermatids; 1Nb:2n = “Onion stage” spermatids with 1 large nebenkern (Nb) associated to 2 haploid nuclei (n); 1Nb:4n = “Onion stage” spermatids with 1 large Nb associated to 4 haploid nuclei; DS = Spermatids with different-sized nuclei; *Df(2R)* = *Df(2R)Exel7138*.

The anti-Spd2 immunostaining of primary spermatocytes at stages S5-S6 (mature primary spermatocytes) revealed that, while wild type primary spermatocytes had two pairs of V-shaped elongated centrioles, most S5-S6 *DmATPCL* primary spermatocytes displayed more than two Spd2-stained foci, indicating premature centriole disengagement (Figure 1Ca). As a consequence, meiotic cells from *DmATPCL* males undergoing meiosis I and II assembled irregular spindles since prophase/metaphase I (MI) (Figure 1Cb). As expected, we have also observed that all multipolar meiotic figures analyzed showed chromosome segregation defects that resulted in lagging chromosomes and eventually led to aneuploid spermatids (Figure 1Cb–d). However, the frequency of aneuploid spermatids in *DmATPCL* mutant testes (Figure 2C) was in general lower than the frequency of multipolar spindles, indicating that most cells with aberrant spindles did not complete meiosis II. Interestingly, we have also observed that a small, yet statistically significant fraction of anaphases I (~6%, n = 80 cells/mutant) from all mutant combinations analyzed, showed apparent normal bipolar spindles, but with daughter nuclei decondensed in a telophase state, indicating that, unlike control cells, central spindles likely failed to constrict during telophase and degenerate (Figure 1Ce). Collectively, these results suggest that DmATPCL is required to maintain a proper centriole attachment during male meiotic prophase and a correct organization of bipolar spindle structures.

### 3.3. Cytokinesis Defects in DmATPCL Mutants

The high frequency of irregular spermatids exhibiting large nebenkerns associated to 2 or 4 nuclei indicated that loss of DmATPCL affects cytokinesis. To investigate whether the cytokinesis failure resulted from mislocalization of key components for *Drosophila* cytokinesis during male meiosis, we first immunostained *DmATPCL^DG23402^/Df(2R)Exel7138* and *DmATPCL^DG23402^/DmATPCL^01466^* for anillin that, by mediating the interactions between central spindle microtubules and the equatorial cortex, is a structural component of the contractile ring [26,27]. In normal primary spermatocytes, anillin starts to concentrate in a ring-shape configuration at the cell equator of dividing spermatocytes during anaphase and constricts in telophase during both meiotic divisions (Figure 2A). We found that in mutant telophases showing unconstrained central spindles, anillin localized at the central spindle to eventually form a contractile ring, failed to constrict and appeared fuzzy or discontinuous (Figure 2Ba–c). This phenotype, which is reminiscent of that observed in several cytokinesis mutants [19] is likely to account for part of mutant spermatids with large nebenkern associated to 2 or 4 haploid nuclei (Figure 1A). In the multipolar spindles from the two *DmATPCL* mutant combinations analyzed, we found that anillin still localized at the equator of cells and participated to the formation of one single contractile ring that eventually completed cytokinesis (Figure 2Bd–f). As expected, daughter cells resulting from these divisions may contain 0 to 4 nuclei depending of the number of centrosomes that have been enclosed during the formation of multipolar spindles (Figure 2B). Thus, most of the irregular spermatids consisting of large nebenkern associated to more than one nucleus likely result from a completed cytokinesis occurred in these aberrant spindles. We have also analyzed the localization of additional factors that are enriched at the central spindle during cytokinesis: Fascetto (Feo), the *Drosophila* ortholog of PRC1, which is one of the first markers that localizes to the central overlap region of the anaphase central spindle in spermatocytes [20,22], and Pavarotti (Pav), the ortholog of the microtubule kinesin MKLP1, which is essential for central spindle assembly in *Drosophila* spermatocytes [17,18]. We found that both proteins exhibited a localization pattern in the mutant spindles similar to that elicited by anillin (Supplementary Figure S2), indicating that cytokinesis defects arising from this multipolar anaphase are not due to loss of contractile ring components.

### 3.4. Defective Fusome Branching in DmATPCL Mutant Spermatocyte Cysts

**Figure 3:**
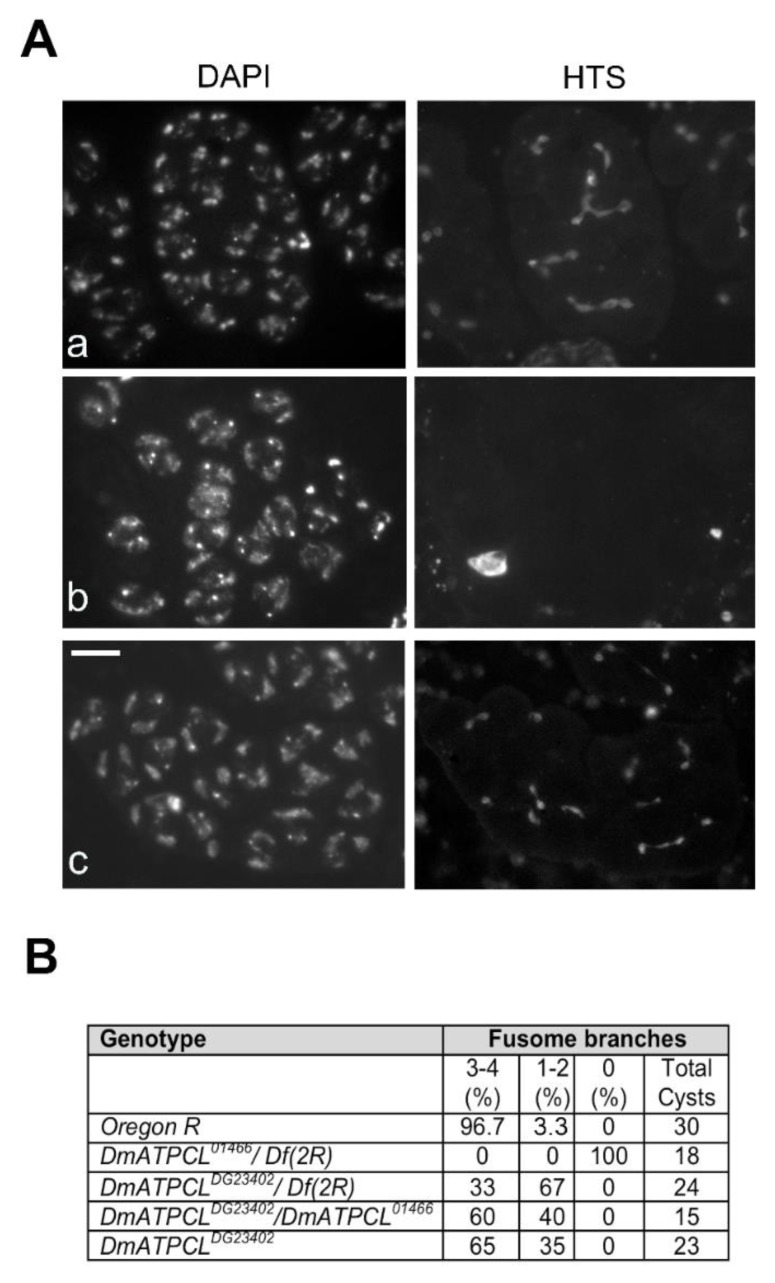
DmATPCL is required for fusome assembly. Hts localization in control (OR) and *DmATPCL* mutant primary spermatocyte cysts. Note that while the fusome elongates and extends clonally towards related germ cells in a wild-type cyst, giving rise to an evident branched structure (**Aa**), *DmATPCL^01466^/Df(2R)* mutant primary spermatocyte cysts are devoid of fusome (**Ab**) and *DmATPCL ^DG23402^/DmATPCL^01466^* cysts display a drastically reduced branching (**Ac**). Note that cysts devoid of fusome, Hts accumulates in distinct and large aggregates. Bar = 10 μm. (**B**) Frequency of mutant cysts showing defective branching of fusome. Percentages refer to S3–S4 (apolar) primary spermatocyte-containing cysts showing the indicated number of fusome branches. *Df(2R)* = *Df(2R)Exel7138.*

Previous work has shown that multipolar spindles and abnormal centriole content in primary spermatocytes are associated with perturbation of male fusome, an ER-derived germline-specific cytoskeleton, which plays an essential role in tethering germ cells within a cyst and in orienting mitotic spindles to achieve synchronized mitoses of cystocytes [21,28,29,30]. We thus checked the organization of male fusome in *DmATPCL* mutant spermatocyte cysts by first analyzing the localization of *hu-li tai-shao* encoding product Hts, an adducine-like protein found associated to the fusome [21,31,32]. The fusome initially arises from a spherical structure, called the spectrosome, in the germline stem cell, elongates and extends clonally towards related germ cells in a cyst, through the ring canals, and finally regresses in mature spermatocytes, breaking down in pieces [29,33]. Our immonostaining with anti-Hts antibody revealed that, whereas wild-type cysts of spermatogonia or primary young spermatocytes exhibited a characteristic developmental pattern of a branched male fusome, almost the totality of *DmATPCL^01466^/Df(2R)* mutant cysts lacked the ribbon like-structures and was mostly present in distinct and large aggregates (Figure 3). Fusome branching was also altered in *DmATPCL ^DG23402^/DmATPCL^01466^* mutant testes, although in these mutants the phenotype was not as aberrant as in *DmATPCL^01466^* and Hts aggregates were never found (Figure 3). Similar pattern was also observed for F-actin by immunostaining spermatocyte cysts with phalloidin that binds F-actin (Supplementary Figure S3). Consistently, our observations indicate that DmATPCL plays also a crucial role in fusome organization in premeiotic cells. The absence of a properly assembled fusome found in *DmATPCL* mutant cysts can partially account for the abnormal behavior of centrioles during spermatogenesis in these mutants. However, we can’t rule out that DmATPCL could play a direct role in regulating the expression or/and the stability of factors required for centrosome dynamics in *Drosophila* such as Polo and Myt kinases [30,34]. It is also plausible that, as shown in human cells, DmATPCL could localize at ER in *Drosophila* spermatocytes and its depletion would thus perturb the organization of ER-derived structures, such as the fusome. However, as we failed to localize DmATPCL by immunofluorescence in meiotic cells even using different fixation procedures, further investigation is required to eventually confirm this issue.

### 3.5. DmATPCL Mutant Phenotype Is a Consequence of Reduced Levels of Fatty Acids

It is widely accepted that ACL-derived Acetyl-CoA, in addition to protein acetylation, is required for lipogenesis and cholesterogenesis in mammals. As *Drosophila*, like other insects, does not synthetize sterols and requires dietary sterols to complete development [35], it is reasonable to envisage that DmATPCL is mainly involved in fatty acid biosynthesis. Thus, we asked whether levels of fatty acids and triglycerides were affected in *DmATPCL ^DG23402^* mutants. To this aim, we measured free fatty acid and triglyceride contents in these mutants and found that although the total amount of triglycerides did not vary considerably, the fatty acids levels were 30% reduced compared to wild-type control (Supplementary Figure S4). This indicates that DmATPCL-derived Acetyl-CoA in flies is mainly required for the generation of fatty acids. We also checked the cholesterol levels and found that *DmATPCL* mutants elicited no reduction of cholesterol levels (Supplementary Figure S4). This was not unexpected, as *Drosophila*, like other invertebrates, cannot synthetize sterols from small carbon units [36]. To verify whether reduction of fatty acids could be taken into account as causative of *DmATPCL ^DG23402^* mutant phenotype, we raised *DmATPCL ^DG23402^* mutants as well as control flies in fatty acid-supplemented fly food and checked testes for the presence of aberrant spermatids. We found that adding fatty acids (0.5% oleic and arachidonic acids) to fly food led to a low, still statistically significant, reduction of the frequency of irregular spermatids resulting from cytokinesis defects in *DmATPCL ^DG23402^* mutants (Supplementary Figure S4), indicating that cytokinesis defects are partially alleviated by dietary fatty acid. Taken together, these results indicate that the reduction of fatty acids could cause defective meiosis. Indeed, as these products are building blocks of several metabolic pathways, it is conceivable that cellular processes that demand high levels of lipogenesis (such as spermatogenesis) are primarily affected by perturbation of DmATPCL function. A broad number of evidence indicate that metabolic regulators of many lipids, including phospathidylinositol (PI) lipids, and fatty acids act as key players during *Drosophila* cytokinesis, as they modulate the dynamics of cytokinesis structure and regulate membrane addition (reviewed in [19]).

We hypothesize that cytokinesis defects seen in spermatids result also from inappropriate resolution of multipolar spindles, which arise from spermatocytes with abnormal centrosomes, are still able to assemble one single contractile ring and ultimately leads to the formation of multinucleate spermatids. Cytokinesis failure has been indeed directly associated to the formation of multipolar spindles in different mutants such as *polo*, *dbf*, and *vib* [34,37,38,39]. In the same mutants, defects in chromosome segregation, similar to those found in *DmATPCL* mutants, were also observed, indicating that irregular centrosome behavior, multipolar spindles, and spermatids with either large nebenkern or micronuclei are tightly linked events.

In conclusion, our study reveals that DmATPCL is involved in male meiosis at different levels. Its involvement in this complex developmental program mainly relies on its enzymatic activity on lipogenesis. Moreover, it is also required for regulating fusome and centriole disengagement by mechanisms that are still unclear. Interestingly, a concomitant effect on fusome and centrioles has been also found as a consequence of depletion of Myt, leaving open and intriguing possibility that both factors could share a relevant functional relationship, which it would be of future interest to examine. Why mutations in *DmATPCL* strongly affect male meiotic division but not mitosis is unclear. This is not an unexpected finding, as several papers have reported examples of many other genes that, when mutated impair meiotic, but not mitotic, *Drosophila* cell divisions, although they are expressed similarly in both germ-line and somatic tissues. One possible explanation is that *Drosophila* testes, differently from brains, require more complex membrane remodeling processes, including rapid spermatocyte growth and cytokinesis (reviewed in [19,40]). Moreover, it has been proven that lipid composition in larval brain is more steadily and independently regulated with respect to remain parts of larval body [41]. Thus, it is conceivable that brains have developed compensatory functions to cope with lipid deficiency.

## Supplementary Materials

The following are available online at https://www.mdpi.com/2073-4409/9/1/206/s1, Figure S1: Analysis of expression of *DmATPCL*, Figure S2: Fascetto (Feo) and Pavarotti (Pav) localization in *DmATPCL* mutant ana-telophase, Figure S3: F-Actin localization in *DmATPCL^01466^* hemizygous testes, Figure S4: Fatty acid levels are reduced upon depletion of *DmATPCL*.

## References

[1] Chypre M., Zaidi N., Smans K. (2012). ATP-citrate lyase: A mini-review. Biochem. Biophys. Res. Commun..

[2] Sivanand S., Rhoades S., Jiang Q., Lee J.V., Benci J., Zhang J., Yuan S., Viney I., Zhao S., Carrer A. (2017). Nuclear Acetyl-CoA Production by ACLY Promotes Homologous Recombination. Mol. Cell.

[3] Wellen K.E., Hatzivassiliou G., Sachdeva U.M., Bui T.V., Cross J.R., Thompson C.B. (2009). ATP-citrate lyase links cellular metabolism to histone acetylation. Science.

[4] Verschueren K.H.G., Blanchet C., Felix J., Dansercoer A., De Vos D., Bloch Y., Van Beeumen J., Svergun D., Gutsche I., Savvides S.N. (2019). Structure of ATP citrate lyase and the origin of citrate synthase in the Krebs cycle. Nature.

[5] Chen Y., Deb D.K., Fu X., Yi B., Liang Y., Du J., He L., Li Y.C. (2019). ATP-citrate lyase is an epigenetic regulator to promote obesity-related kidney injury. FASEB J..

[6] Zhong R., Cui D., Richardson E.A., Phillips D.R., Azadi P., Lu G., Ye Z.H. (2019). Cytosolic Acetyl-CoA generated by ATP-citrate lyase is essential for acetylation of cell wall polysaccharides. Plant. Cell Physiol..

[7] Zaidi N., Royaux I., Swinnen J.V., Smans K. (2012). ATP citrate lyase knockdown induces growth arrest and apoptosis through different cell- and environment-dependent mechanisms. Mol. Cancer Ther..

[8] Zaidi N., Swinnen J.V., Smans K. (2012). ATP-citrate lyase: A key player in cancer metabolism. Cancer Res..

[9] Bauer D.E., Hatzivassiliou G., Zhao F., Andreadis C., Thompson C.B. (2005). ATP citrate lyase is an important component of cell growth and transformation. Oncogene.

[10] Hatzivassiliou G., Zhao F., Bauer D.E., Andreadis C., Shaw A.N., Dhanak D., Hingorani S.R., Tuveson D.A., Thompson C.B. (2005). ATP citrate lyase inhibition can suppress tumor cell growth. Cancer Cell.

[11] Migita T., Okabe S., Ikeda K., Igarashi S., Sugawara S., Tomida A., Soga T., Taguchi R., Seimiya H. (2014). Inhibition of ATP citrate lyase induces triglyceride accumulation with altered fatty acid composition in cancer cells. Int. J. Cancer.

[12] Morciano P., Di Giorgio M.L., Porrazzo A., Licursi V., Negri R., Rong Y., Cenci G. (2019). Depletion of ATP-Citrate Lyase (ATPCL) Affects Chromosome Integrity without Altering Histone Acetylation in Drosophila Mitotic Cells. Front. Physiol..

[13] Peleg S., Feller C., Forne I., Schiller E., Sevin D.C., Schauer T., Regnard C., Straub T., Prestel M., Klima C. (2016). Life span extension by targeting a link between metabolism and histone acetylation in Drosophila. EMBO Rep..

[14] Bonaccorsi S., Giansanti M.G., Cenci G., Gatti M. (2012). F-actin staining of Drosophila testes. Cold Spring Harb. Protoc..

[15] Bonaccorsi S., Giansanti M.G., Cenci G., Gatti M. (2011). Methanol-acetone fixation of Drosophila testes. Cold Spring Harb. Protoc..

[16] Bonaccorsi S., Giansanti M.G., Cenci G., Gatti M. (2012). Formaldehyde fixation of Drosophila testes. Cold Spring Harb. Protoc..

[17] Adams R.R., Tavares A.A., Salzberg A., Bellen H.J., Glover D.M. (1998). pavarotti encodes a kinesin-like protein required to organize the central spindle and contractile ring for cytokinesis. Genes Dev..

[18] Carmena M., Riparbelli M.G., Minestrini G., Tavares A.M., Adams R., Callaini G., Glover D.M. (1998). Drosophila polo kinase is required for cytokinesis. J. Cell Biol..

[19] Giansanti M.G., Fuller M.T. (2012). What Drosophila spermatocytes tell us about the mechanisms underlying cytokinesis. Cytoskeleton.

[20] Verni F., Somma M.P., Gunsalus K.C., Bonaccorsi S., Belloni G., Goldberg M.L., Gatti M. (2004). Feo, the Drosophila homolog of PRC1, is required for central-spindle formation and cytokinesis. Curr. Biol..

[21] Wilson P.G. (2005). Centrosome inheritance in the male germ line of Drosophila requires hu-li tai-shao function. Cell Biol. Int..

[22] Giansanti M.G., Vanderleest T.E., Jewett C.E., Sechi S., Frappaolo A., Fabian L., Robinett C.C., Brill J.A., Loerke D., Fuller M.T. (2015). Exocyst-Dependent Membrane Addition Is Required for Anaphase Cell Elongation and Cytokinesis in *Drosophila*. PLoS Genet..

[23] Dix C.I., Raff J.W. (2007). Drosophila Spd-2 recruits PCM to the sperm centriole, but is dispensable for centriole duplication. Curr. Biol..

[24] Giansanti M.G., Bucciarelli E., Bonaccorsi S., Gatti M. (2008). Drosophila SPD-2 is an essential centriole component required for PCM recruitment and astral-microtubule nucleation. Curr. Biol..

[25] Cenci G., Bonaccorsi S., Pisano C., Verni F., Gatti M. (1994). Chromatin and microtubule organization during premeiotic, meiotic and early postmeiotic stages of Drosophila melanogaster spermatogenesis. J. Cell Sci..

[26] D’Avino P.P., Takeda T., Capalbo L., Zhang W., Lilley K.S., Laue E.D., Glover D.M. (2008). Interaction between Anillin and RacGAP50C connects the actomyosin contractile ring with spindle microtubules at the cell division site. J. Cell Sci..

[27] Gregory S.L., Ebrahimi S., Milverton J., Jones W.M., Bejsovec A., Saint R. (2008). Cell division requires a direct link between microtubule-bound RacGAP and Anillin in the contractile ring. Curr. Biol..

[28] Hime G.R., Brill J.A., Fuller M.T. (1996). Assembly of ring canals in the male germ line from structural components of the contractile ring. J. Cell Sci..

[29] Gunsalus K.C., Bonaccorsi S., Williams E., Verni F., Gatti M., Goldberg M.L. (1995). Mutations in twinstar, a Drosophila gene encoding a cofilin/ADF homologue, result in defects in centrosome migration and cytokinesis. J. Cell Biol..

[30] Varadarajan R., Ayeni J., Jin Z., Homola E., Campbell S.D. (2016). Myt1 inhibition of Cyclin A/Cdk1 is essential for fusome integrity and premeiotic centriole engagement in Drosophila spermatocytes. Mol. Biol. Cell.

[31] Yue L., Spradling A.C. (1992). hu-li tai shao, a gene required for ring canal formation during Drosophila oogenesis, encodes a homolog of adducin. Genes Dev..

[32] Zaccai M., Lipshitz H.D. (1996). Role of Adducin-like (hu-li tai shao) mRNA and protein localization in regulating cytoskeletal structure and function during Drosophila Oogenesis and early embryogenesis. Dev. Genet..

[33] Giansanti M.G., Bonaccorsi S., Gatti M. (1999). The role of anillin in meiotic cytokinesis of Drosophila males. J. Cell Sci..

[34] Herrmann S., Amorim I., Sunkel C.E. (1998). The POLO kinase is required at multiple stages during spermatogenesis in *Drosophila melanogaster*. Chromosoma.

[35] Cooke J., Sang J.H. (1970). Utilization of sterols by larvae of *Drosophila melanogaster*. J. Insect Physiol..

[36] Clayton R.B. (1964). The Utilization of Sterols by Insects. J. Lipid Res..

[37] Riparbelli M.G., Gottardo M., Glover D.M., Callaini G. (2014). Inhibition of Polo kinase by BI2536 affects centriole separation during Drosophila male meiosis. Cell Cycle.

[38] Gatt M.K., Glover D.M. (2006). The Drosophila phosphatidylinositol transfer protein encoded by vibrator is essential to maintain cleavage-furrow ingression in cytokinesis. J. Cell Sci..

[39] Sechi S., Frappaolo A., Karimpour-Ghahnavieh A., Gottardo M., Burla R., Di Francesco L., Szafer-Glusman E., Schinina E., Fuller M.T., Saggio I. (2019). Drosophila Doublefault protein coordinates multiple events during male meiosis by controlling mRNA translation. Development.

[40] Bonaccorsi S., Gatti M. (2017). Drosophila Male Meiosis. Methods Mol. Biol.

[41] Carvalho M., Schwudke D., Sampaio J.L., Palm W., Riezman I., Dey G., Gupta G.D., Mayor S., Riezman H., Shevchenko A. (2010). Survival strategies of a sterol auxotroph. Development.

