# Supplementary material for: The Drosophila Citrate Lyase Is Required for Cell Division during Spermatogenesis
Source: Cells. 2020 Jan 14;9(1):206. doi: 10.3390/cells9010206 (PMC7016701; doi:10.3390/cells9010206)
Supplement: Supplementary file 1 [file cells-09-00206-s001.pdf]

The *Drosophila* Citrate Lyase is Required for Cell Division during Spermatogenesis

Maria Laura Di Giorgio, Patrizia Morciano, Elisabetta Bucciarelli, Antonella Porrazzo, Francesca Cipressa, Sara Saraniero, Diana Manzi, Yikang S. Rong and Giovanni Cenci

Supplementary Material

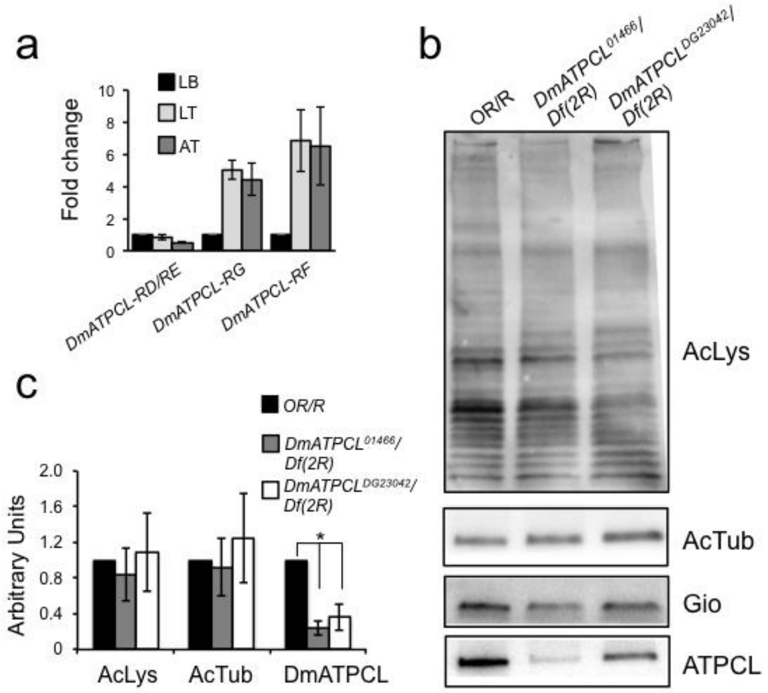

Figure S1. Analysis of expression of *DmATPCL*. **a**) qPCR from wild-type larval brain (LB), larval testes (LT) and adult testes (AT) showing the expression of *-RD, -RE, -RF* and *–RG* transcripts encoded by *DmATPCL;* **b**) Western Blot from control (OR/R) and *DmATPCL* mutant hemizygous testes with anti-ATPCL,-AcLys and –AcTub antibodies showing that general protein acetylation is not reduced even in the strongest *DmATPCL*^{01466} hemizygous mutants. Anti-Giotto (Gio) has been used as a loading control. c) Quantification of AcLys, AcTub and DmATPCL protein levels. Three independent replicates were used for this quantification. * (p < 0.05; t-Student test).

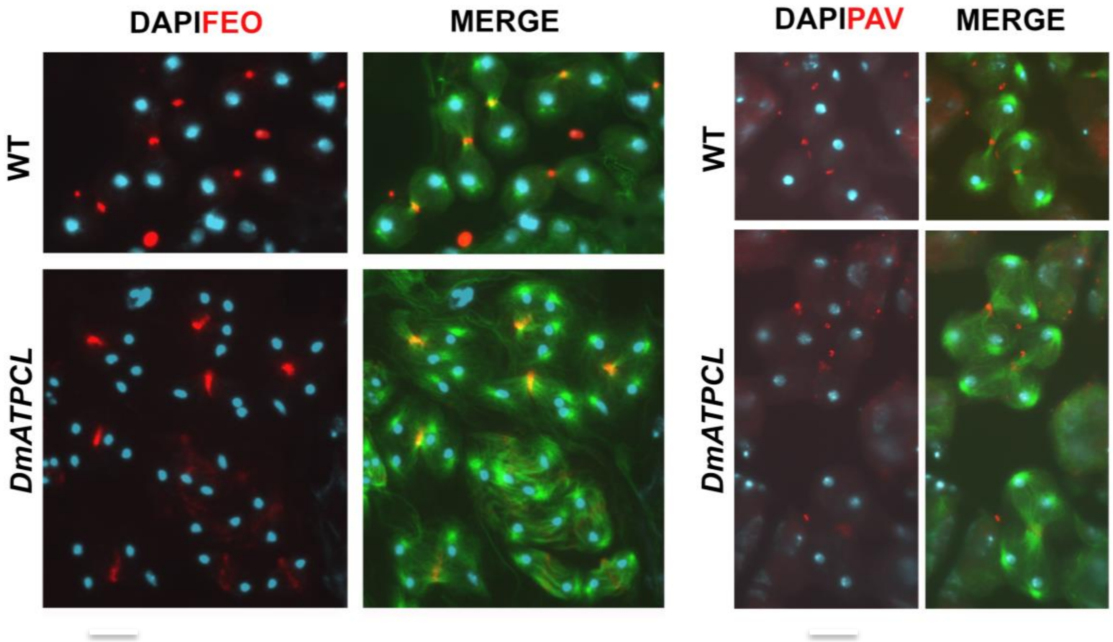

Figure S2. Fascetto (Feo) and Pavarotti (Pav) localization in *DmATPCL* mutant ana-telophase. Note that, like anillin, the PRC1 ortholog Feo and the MKLP1-like microtubule kinesin Pavarotti (Pav) are able to localize in the cell midzones of *DmATPCL* mutant multipolar spindles as revealed in the merge with tubulin (green). Bar: 10 µm.

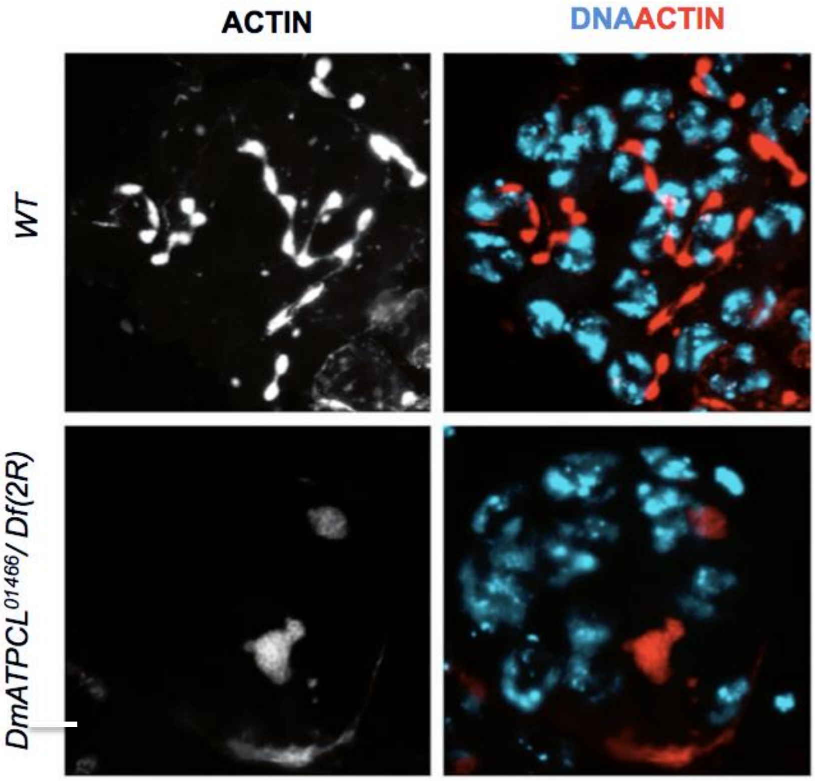

Figure S3. F-Actin localization in *DmATPCL*^{01466} hemizygous testes. Note that, whereas F-actin, like Hts, normally decorates the fusome branched structure in wild-type primary spermatocyte cysts, it forms a large aggregate in *DmATPCL* mutants. Bar: 5 µm.

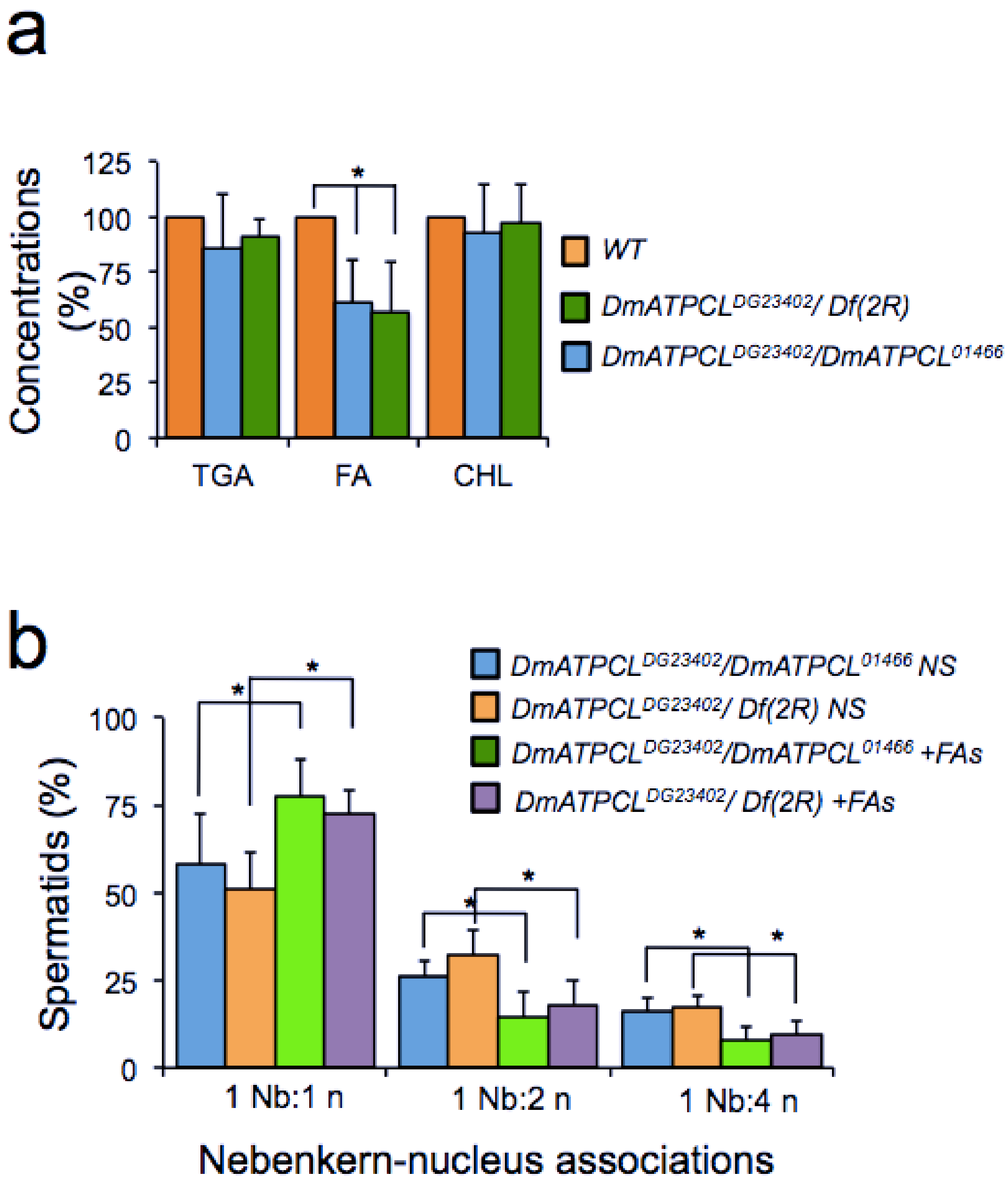

Figure S4. Fatty acid levels are reduced upon depletion of *DmATPCL*. **a**) Concentrations of Fatty Acids (FA), Triglycerides (TGA) and Cholesterol (CHL) in different *DmATPCL* mutant combinations. Note that only FA levels drop as a consequence of depletion of DmATPCL); **b**) Frequency of irregular spermatids in *DmATPCL* mutant testes upon supplementation of Fatty Acids (+FAs). See text for further details. (* $p < 0.05$; *t*-Student test).
